# Unveiling the Potential of CuO and Cu_2_O Nanoparticles against Novel Copper-Resistant *Pseudomonas* Strains: An In-Depth Comparison

**DOI:** 10.3390/nano14201644

**Published:** 2024-10-13

**Authors:** Olesia Havryliuk, Garima Rathee, Jeniffer Blair, Vira Hovorukha, Oleksandr Tashyrev, Jordi Morató, Leonardo M. Pérez, Tzanko Tzanov

**Affiliations:** 1 Department of Extremophilic Microorganisms Biology, D. K. Zabolotny Institute of Microbiology and Virology of the National Academy of Sciences of Ukraine, 154 Zabolotny St., 03143 Kyiv, Ukraine or olesia.havryliuk@upc.edu (O.H.); or vira.hovorukha@uni.opole.pl (V.H.); or oleksandr.tashyrev@uni.opole.pl (O.T.); 2 Laboratory of Sanitary and Environmental Microbiology (MSMLab), UNESCO Chair on Sustainability, Universitat Politècnica de Catalunya-BarcelonaTech (UPC), Rambla de Sant Nebridi 22, 08222 Terrassa, Barcelona, Spain; jordi.morato@upc.edu (J.M.); leonardo.martin.perez@upc.edu (L.M.P.); 3 Grup de Biotecnologia Molecular i Industrial, Departament d’Enginyeria Química, Universitat Politècnica de Catalunya-BarcelonaTech (UPC), Rambla de Sant Nebridi 22, 08222 Terrassa, Barcelona, Spain; garima.rathee@upc.edu (G.R.); jeniffer.blair@upc.edu (J.B.); 4 Institute of Environmental Engineering and Biotechnology, University of Opole, 45-040 Opole, Poland

**Keywords:** copper oxide nanoparticles, sonochemical synthesis, metal-resistant bacteria, *Pseudomonas* sp., wild-type bacteria control

## Abstract

Four novel *Pseudomonas* strains with record resistance to copper (Cu^2+^) previously isolated from ecologically diverse samples (*P. lactis* UKR1, *P. panacis* UKR2, *P. veronii* UKR3, and *P. veronii* UKR4) were tested against sonochemically synthesised copper-oxide (I) (Cu_2_O) and copper-oxide (II) (CuO) nanoparticles (NPs). Nanomaterials characterisation by X-ray diffractometry (XRD), X-ray photoelectron spectroscopy (XPS), Fourier transform infrared spectroscopy (FTIR), and High-Resolution Transmission Electron Microscopy (HRTEM) confirmed the synthesis of CuO and Cu_2_O NPs. CuO NPs exhibited better performance in inhibiting bacterial growth due to their heightened capacity to induce oxidative stress. The greater stability and geometrical shape of CuO NPs were disclosed as important features associated with bacterial cell toxicity. SEM and TEM images confirmed that both NPs caused membrane disruption, altered cell morphology, and pronounced membrane vesiculation, a distinctive feature of bacteria dealing with stressor factors. Finally, Cu_2_O and CuO NPs effectively decreased the biofilm-forming ability of the Cu^2+^-resistant UKR strains as well as degraded pre-established biofilm, matching NPs’ antimicrobial performance. Despite the similarities in the mechanisms of action revealed by both NPs, distinctive behaviours were also detected for the different species of wild-type *Pseudomonas* analysed. In summary, these findings underscore the efficacy of nanotechnology-driven strategies for combating metal tolerance in bacteria.

## 1. Introduction

The investigation of microorganisms within the *Pseudomonas* genus has remained pertinent and enduring over the years. Pseudomonads, owing to their widespread distribution across different natural and man-made ecosystems, have adapted to diverse environmental conditions, including extreme ones. They are characterised as highly resistant microorganisms exhibiting resilience against both biotic and abiotic factors [1,2,3]. Moreover, *Pseudomonas* sp. are well known for being capable of resisting and degrading highly toxic compounds (e.g., heavy metals, fuel oils and oil-derived by-products, antibiotics, pesticides, harmful chemicals) in polluted soil and water [1]. Despite these promising environmental applications, some *Pseudomonas* species are opportunistic pathogens and can cause severe infections in living organisms (including humans), such as pseudomonosis, bacteraemia, and biofilm-associated infections, among others [4,5,6,7]. Therefore, new *Pseudomonas* isolates must be thoroughly studied, especially those strains with internal and acquired antimicrobial resistance.

During the last few years, an increasing interest in the development of nano-inspired solutions to control antimicrobial-resistant bacteria has been explored [8,9,10,11,12]. However, microorganisms can also adapt to nanomaterial toxicity. In particular, metal-based nanoparticles (NPs) have demonstrated potent antibacterial properties, at low concentrations, against resistant microorganisms [13,14,15,16,17]. Among them, metallic copper NPs and copper oxide NPs have effectively inhibited fungal, viral, and bacterial activity [18,19,20,21,22]. Additionally, the increasing interest in copper oxide NPs stems from their comparatively lower cost of production compared to other metal-based NPs, such as those containing silver or gold [18]. Recent studies have suggested that copper oxide NPs possess high surface reactivity and the ability to diffuse directly through the cellular membrane [23], triggering the production of reactive oxygen species (ROS), which damage both bacterial proteins and nucleic acids [19]. However, few studies [24,25,26] have been conducted so far to analyse the effect of copper-containing NPs on microorganisms within the *Pseudomonas* genus other than the model organism *P. aeruginosa* [27,28]. No studies have focused on analysing the antimicrobial performance of metal-based NPs against metal-resistant microorganisms.

In a previous work, four novel *Pseudomonas* copper-resistant strains (*P. lactis* UKR1, *P. panacis* UKR2, *P. veronii* UKR3, and *P. veronii* UKR4) were isolated from soil samples collected in Ukraine, the Arctic, and the Antarctic ice environments [29]. These bacteria possess the ability to accumulate Cu(II) as well as precipitate the metal when grown on nutrient agar plates amended with high concentrations of copper salts. After sequencing, the in silico analysis of the UKR strains genome revealed the presence of distinctive metal resistance determinants and antibiotic-resistant genes. Both features are concerning since the simultaneous occurrence of resistance to antibiotics and heavy metals is a potential threat to human health and the environmental balance. Later, an in vitro screening of all four strains confirmed the ability of these wild-type isolates to grow in the presence of high concentrations of copper sulphate and copper citrate, as well as the ability to survive and reproduce in the presence of amoxicillin and ceftazidime. Notably, these strains also belong to *Pseudomonas* species that have been underresearched. For instance, *P. panacis* and *P. lactis* were first mentioned in reports from 2005 [30] and 2017 [31], respectively. Hence, there is a gap in our understanding of the metabolic behaviour of these newly discovered *Pseudomonas* species to various stressors, especially when compared to well-studied pseudomonads such as *P. putida*, *P. aeruginosa*, or *P. fluorescence*. Additionally, given the resistance of the novel UKR strains to toxic metals and antibiotics, it is prudent to explore green approaches for their control.

Considering this, the present study examines the effectiveness of sonochemically synthesised antimicrobial copper oxide (I) (Cu_2_O) and copper oxide (II) (CuO) NPs on the growth, cell morphological alterations, and biofilm formation ability of the copper-resistant UKR strains. Furthermore, this work discusses and compares some of the proven mechanisms of action of both types of copper oxide NPs and how these wild-type UKR strains could potentially mitigate NP toxicity.

## 2. Materials and Methods

### 2.1. Synthesis of CuO NPs

The CuO NPs were synthesised by using copper (II) acetate monohydrate (Cu(CH_3_COO)_2_·H_2_O) (Sigma-Aldrich, Barcelona, Spain) and sodium hydroxide (NaOH, Sigma-Aldrich, Barcelona, Spain) as precursors. A solution containing (Cu(CH_3_COO)_2_·H_2_O) (0.01 M, 20 mL) was introduced into a 100 mL sonication glass vessel maintained at 60 °C. Subsequently, NaOH (5 M, 75 µL) was added to the solution, and the resulting mixture was promptly subjected to sonication in an ultrasonic horn (VCX750 Sonics Vibra-Cell^TM^, Sonics & Materials, Inc., Newtown, CT, USA) operating at 20 kHz and 50% amplitude for 5 min. Later, the NPs were collected via centrifugation at 4000× *g* for 15 min. The obtained NPs were then washed with deionised water to remove impurities before undergoing lyophilisation to yield solid CuO NPs.

### 2.2. Synthesis of Cu_2_O NPs

Cu_2_O NPs were synthesised utilising copper (II) acetate monohydrate (Cu(CH_3_COO)_2_·H_2_O) and glycerol (Sigma-Aldrich, Barcelona, Spain) as the primary precursors. In a standard synthesis procedure, (Cu(CH_3_COO)_2_·H_2_O) (0.5 g) and glycerol (15 mL) were introduced into a 100 mL sonication glass vessel pre-heated at 60 °C. Subsequently, the solution underwent ultrasonication using an ultrasonic horn (VCX750 Sonics Vibra-Cell^TM^, Sonics & Materials, Inc., Newtown, CT, USA) operating at 20 kHz and 50% amplitude for 1 h. The solution’s colour changed from blue to brick-red during the reaction, indicating the formation of Cu_2_O NPs. Post-sonication, the reaction mixture was diluted with deionised water, followed by NP collection through centrifugation (4000× *g*, 15 min). The collected Cu_2_O NPs were further washed with deionised water and lyophilised to yield solid Cu_2_O NPs [32].

### 2.3. Characterisation of CuO and Cu_2_O NPs

The X-ray diffraction (XRD) patterns were obtained using a Bruker D8 Advance diffractometer (Bruker Inc., Billerica, MA, USA). The X-ray photoelectron spectroscopy (XPS) analyses were conducted in the *Centre de Recerca en Ciència i Enginyeria Multiescala de Barcelona* (Universitat Politècnica de Catalunya, Sant Adrià de Besòs, Spain) in a SPECS system with a PHOIBOS 150 EP hemispherical energy analyser equipped with a MCD-9 detector. An X-ray source Mg Kα line of 1253.4 eV energy and 100 W power was used at a pass energy of 20 eV, placed at 54° in respect to the analyser axis and calibrated by the 3d5/2 line of silver (Ag) with a full width at half maximum (FWHM) of 1.211 eV. Survey spectra were recorded at an energy step of 1 eV and high-resolution spectra of 0.1 eV. Morphological studies were conducted by transmission electron microscopy (TEM) by casting 10 μL of diluted samples onto copper grids (holey carbon films) and air-dried for 15 min. The TEM images were recorded using a FEI Tecnai G2 F20 high-resolution microscope equipped with a 200 kV field emission gun (FEI Inc., Valley City, ND, USA). Fourier transform infrared (FTIR) spectra were obtained in a PerkinElmer Spectrum 100 spectrometer (PerkinElmer^TM^, Waltham, MA, USA) equipped with an attenuated total reflection (ATR) component of germanium crystal featuring a high-resolution index (4.0) by conducting 64 scans per sample at 4 cm^−1^ resolution. UV-visible spectra were recorded on a Varian Cary 100 Bio spectrophotometer (Varian Inc., Belrose, Australia).

### 2.4. Bacterial Strains

Four wild-type strains of *Pseudomonas* identified and named as *P. lactis* UKR1, *P. panacis* UKR2, *P. veronii* UKR3, and *P. veronii* UKR4 were tested. The strains were previously isolated from natural soil samples and characterised as copper-resistant bacteria. Additionally, the genomes of the four new strains were sequenced and analysed, showing variations in the presence of genes encoding for copper resistance (i.e., *cop*A, *cop*B, *cop*C, *cop*D, *cus*RS), among other genes associated with heavy metal efflux systems and antibiotic resistance [29]. All four strains can grow in the presence of high concentrations of copper sulphate (Figure S1, Supplementary Material).

### 2.5. Antimicrobial Properties of CuO and Cu_2_O NPs

#### 2.5.1. Growth Curve Assays

The effect of CuO and Cu_2_O NPs on UKR strain growth was determined by measuring the optical density recorded at 600 nm (OD_600_) during 48 h at 35 °C in a TECAN Infinite M200 Microplate Reader (GENios-Tecan, Männedorf, Switzerland) [33]. The growth curves were performed in Luria-Bertani (LB) broth in the absence (control) or presence of 50 and 100 mg/L of NPs. The concentrations were selected considering the results from Figure S1 (Supplementary Material) and in accordance with previously reported data about CuO and Cu_2_O NPs cytotoxicity against different pathogens, including *Pseudomonas* strains [18,23,24,25,26,27,28]. Fresh overnight (16–18 h) cultures of *P. lactis* UKR1, *P. panacis* UKR2, *P veronii* UKR3, and *P veronii* UKR4 were adjusted to an OD_600_ ≈ 0.6–0.7 in sterile saline solution (NaCl 0.85% *w*/*v*) and used as test inoculum (10 μL) for the assays conducted in a sterile 96-well microtiter plate (Sarstedt, Nümbrecht, Germany) with a final volume of 250 μL. The experiments were repeated twice in four replicates.

#### 2.5.2. ROS Measurement

The generation of ROS by bacteria in contact with CuO and Cu_2_O NPs was studied using the oxidation-sensitive probe 2′,7′-dichlorofluorescin diacetate (H_2_DCFDA, Invitrogen, Barcelona, Spain). For the assay, the tested *Pseudomonas* strains (i.e., *P. lactis* UKR1, *P. panacis* UKR2, *P. veronii* UKR3, and *P. veronii* UKR4) were grown in LB broth (Sigma-Aldrich, Spain) for 24 h (35 °C, 230 rpm). Then, the cultures were centrifuged at 8000× *g* for 5 min at room temperature and the cells resuspended in sterile saline solution (0.85% *w*/*v* NaCl) to reach an OD_600_ ≈ 0.6–0.7. Afterwards, 150 µL of each copper NP at 50 and 100 mg/L were mixed with 3 μL of 2 mM H_2_DCFDA solution (prepared in DMSO) directly in the 96-well plate. Finally, 150 μL of bacterial inoculum was added to the reaction mixture, and the plate was incubated in the multiplate reader set at 35 °C. The fluorescence was measured at λ*_ex_*/*_em_* = 490/520 nm for 60 min in a TECAN Infinite M200 Microplate Reader (GENios-Tecan, Männedorf, Switzerland). Different controls were also processed (i.e., bacterial cells without NPs addition, bacterial cells in the presence of NPs but without the addition of H_2_DCFDA, and sterile saline solution with the addition of NPs and H_2_DCFDA). Results were informed as percent changes (%) in the fluorescence values at 60 min relative to the control condition (i.e., bacterial cells without NP addition).

#### 2.5.3. Anti-Biofilm Formation Assay

The biofilm-inhibition assay was carried out using the classical spectrophotometric approach based on crystal violet staining [31]. Bacterial strains were grown in LB broth in the absence (control) or the presence of CuO and Cu_2_O NPs at different concentrations (50 and 100 mg/L) in a sterile 96-well microtiter plate (Sarstedt, Nümbrecht, Germany). Fresh overnight cultures of the tested bacteria growth in LB broth and adjusted to an OD_600_ ≈ 0.6–0.7 in sterile saline solution were used as the test inoculum (10 µL) for the assay. The plate was incubated for 24 h at 35 °C in a culture chamber. After incubation, the medium was removed along with the planktonic bacteria, and the attached cells were washed four times with sterile distilled water. Furthermore, the plate was inverted, gently tapped to remove excess liquid, and dried for 30 min at 35 °C in a culture chamber. Afterwards, 250 µL of a 0.1% (*w*/*v*) crystal violet (Sigma-Aldrich, Barcelona, Spain) staining solution prepared in MilliQ water was added to each well and incubated at room temperature for 15 min. After staining, the cells in the wells were washed four times with 400 mL of sterile distilled water, and the plates were inverted and placed on a paper towel inside a culture chamber at 35 °C for drying during another 30 min. Furthermore, crystal violet extraction was performed by adding 250 µL of 30% *v*/*v* acetic acid to each well and leaving it to incubate for 5 min at room temperature. Finally, 200 µL of the clear solution was transferred to a new 96-well plate, and the absorbance was measured at 550 nm in a TECAN Infinite M200 Microplate Reader (GENios-Tecan, Männedorf, Switzerland). The assay was repeated twice in four replicates.

#### 2.5.4. Biofilm Degradation Assay

The anti-biofouling activity (i.e., biofilm degradation) of CuO and Cu_2_O NPs against wild-type copper-resistant strains *P. lactis* UKR1, *P. panacis* UKR2, *P. veronii* UKR3, and *P. veronii* UKR4 was studied as described by [34] with some modifications. Briefly, the tested bacteria were grown in LB broth (without NPs) in a 96-well microtiter plate for 48 h at 35 °C in a culture chamber to allow biofilm establishment. After incubation, the planktonic cells were carefully removed by pipetting before being replaced with 250 μL of 50 or 100 mg/L CuO or Cu_2_O NPs solutions prepared in sterile PBS buffer. In addition, a sterile PBS buffer was used as the control. Later, the plate was incubated in a culture chamber at 35 °C for another 24 h. Then, the buffer was removed, and the remaining attached cells were washed two times with 400 mL of sterile distilled buffer. Furthermore, the plate was inverted, gently tapped to remove excess liquid, and dried for 30 min at 35 °C. Finally, 250 µL of a 0.1% (*w*/*v*) crystal violet (Sigma-Aldrich, Barcelona, Spain) staining solution was used to reveal the biofilms, as previously described in Section 2.5.3. Two hundred microlitre (200 µL) aliquots of the clear violet solution were transferred to a new 96-well plate, and the absorbance was finally measured at 550 nm using a TECAN Infinite M200 Microplate Reader (GENios-Tecan, Männedorf, Switzerland). The assay was repeated twice in four replicates.

### 2.6. SEM and TEM Analysis of CuO and Cu_2_O NPs’ Interaction with Bacteria

To visualise the effect of both synthesised NPs on the microbial cells, two different microscopic techniques were carried out using the copper-resistant *P. lactis* UKR1 as the model strain. The interaction of CuO and Cu_2_O NPs with the test microorganism was evaluated by scanning electron microscopy (SEM) and TEM [35]. The first step was to pre-cultivate *P. lactis* UKR1 in LB medium for 12 h (OD_600_ ≈ 0.6–0.7). After centrifugation, the supernatant was discarded, and the bacterial cells were treated with the same volume (50 mL) of 100 mg/L of CuO or Cu_2_O NPs in PBS, followed by incubation for 3 h at 37 °C with shaking (250 rpm). To perform the SEM, each sample (3 mL) was filtered immediately after the treatment using a sterile cellulose membrane filter with a pore size of 0.22 µm (Ø 25 mm, Merck Millipore, Guyancourt, France). Later, the filter containing the bacterial cells was fixed with a solution containing 2.5% (*v*/*v*) glutaraldehyde in 0.1 M PBS (pH = 7.4) and stored at 4 °C until further processing. For TEM, each sample (15 mL) was centrifuged at 8000× *g* for 5 min at room temperature to precipitate the cells. After discarding the supernatant, the cells were fixed with paraformaldehyde (2%)/glutaraldehyde (2.5%) solution (in 0.1 M PBS, pH = 7.4) and stored at 4 °C until further processing. Additionally, SEM and TEM imaging of *P. lactis* UKR1 cells without any treatment were obtained and used as the control. All the pre-prepared samples were further processed by the *Servei de Microscòpia i Difracció de Raigs X* (Universitat Autònoma de Barcelona, Cerdanyola del Vallès, Spain) and analysed using a SEM Zeiss Merlin (Carl Zeiss AG, Oberkochen, Germany) microscope and a TEM Hitachi H-7000 (100 kV, Hitachi Ltd., Tokyo, Japan) microscope, respectively.

### 2.7. Data Analysis

Data were reported as the mean value ± standard deviation (S.D.). The ANOVA test was used to compare the effect of Cu_2_O or CuO NPs and their concentrations on the different bacterial parameters evaluated using the SigmaStat 3.5 program (Systat Software Inc., San Jose, CA, USA) at a confidence level of 95%. When the differences between the measured values were statistically significant (*p* < 0.05), the Tukey’s honest significant difference (HSD) post hoc test was for intergroup comparisons.

## 3. Results and Discussion

### 3.1. Characterisation of CuO and Cu_2_O NPs

In nanotechnology, the synthesis of NPs with controlled morphology using green approaches and simple experimental procedures is still a challenging task. Among the different methods used for the development of copper-oxide NPs, the waterborne sonochemical synthesis stands out due to numerous advantages [36]. The use of high-intensity ultrasound provides high energy and fast reaction kinetics, avoiding the use of elevated temperatures, pressures, or long reaction times. In addition, several studies have explored this method to produce Cu_2_O NPs [32,37,38,39,40,41]. A schematic representation of the sonochemical production of CuO and Cu_2_O NPs in our work is shown in Figure S2 (Supplementary Material). Here, we only used two reagents for each NPs synthesis, i.e., copper acetate as a precursor and glycerol or sodium hydroxide. Glycerol, a greener solvent, acts as the reducing and capping agent during the synthesis of Cu_2_O NPs [32].

**Figure 1:**
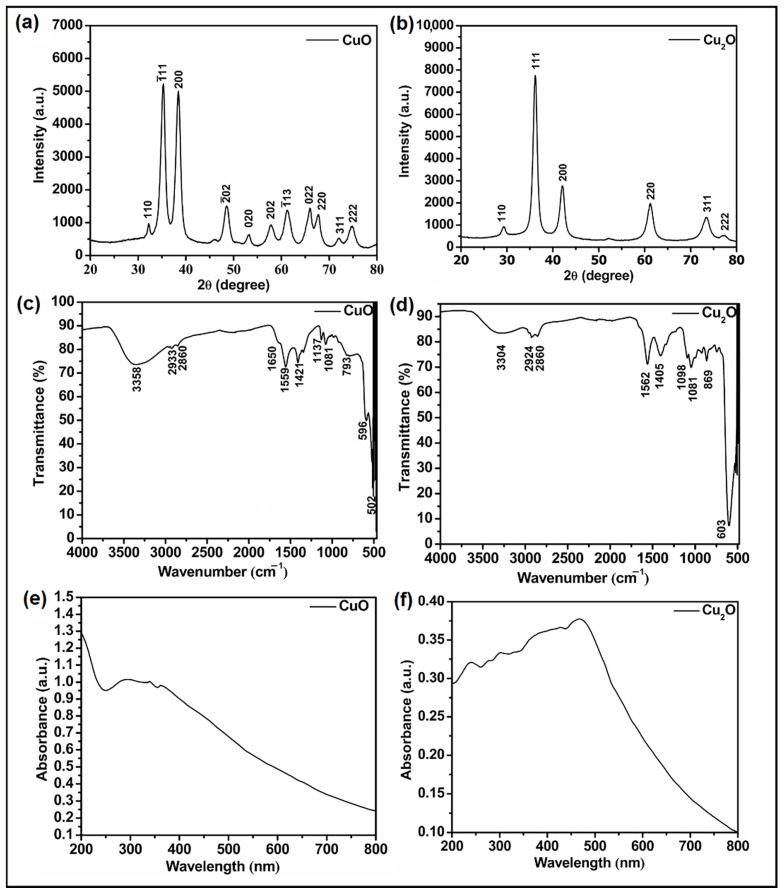
XRD spectra of CuO (**a**) and Cu_2_O NPs (**b**), FTIR spectra of CuO (**c**) and Cu_2_O NPs (**d**), and UV-vis spectra of CuO (**e**) and Cu_2_O NPs (**f**).

The XRD pattern of CuO NPs (Figure 1a) provides information about their crystal phases and crystallinity. The observed diffraction peaks are found to be in a close correlation with previously reported spectral and JCPDS data (48-1548) of CuO NPs [42]. The obtained Miller indices (hkl = (110), $(\bar{1}$11), (210), (200), ($\bar{1}$02), (020), (202), ($\bar{1}$13), (022), (220), (311), (222), and (322)) for the different diffraction peaks confirmed the construction of single-phase CuO NPs with monoclinic structure. The 2θ values with corresponding Miller indices and d-spacing values are summarised in Table S1 (Supplementary Material). Moreover, no extra characteristic peaks were observed corresponding to impurities such as Cu_2_O, Cu(OH)_2_, or precursors, confirming the production of pure CuO NPs.

On the other hand, the observed diffraction peaks in the XRD pattern of Cu_2_O NPs (Figure 1b) are in close correlation with JCPDS data (05-0667) [43] and are summarised along with their Miller indices and d-spacing in Table S1 (Supplementary Material). The observed Miller indices (hkl = (110), (111), (200), (220), (311), and (222)) corresponding to different diffraction peaks confirmed the formation of Cu_2_O NPs. No extra peaks were observed corresponding to the precursors, supporting the synthesis of pure Cu_2_O NPs.

Furthermore, XPS was used to evaluate the oxidation states and elemental composition of Cu_2_O and CuO NPs (Figure S3, Supplementary Material). The high-resolution XPS scan revealed the presence of copper (Cu2p) on the surface of both nanomaterials. For Cu_2_O NPs, the spectra for the Cu2p area displayed the photoelectron peaks at 931.67 and 931.59 eV correlating with the Cu2p (3/2) and Cu2p (Cu (I)), respectively, coupled with a sequence of shakeup satellite peaks at elevated binding energies, associated with Cu2p. Cu^+^ and a small Cu^2+^ state indicated partial oxidation of Cu_2_O, despite Cu^+^ being the predominant species, as anticipated for Cu_2_O NPs [32]. The appearance of Cu^2+^ could also be due to unwanted partial oxidation on the surface of Cu_2_O NPs during sample processing for XPS analysis [44]. For CuO NPs, the presence of the primary peaks at 927.31 and 927 eV corresponds to Cu2p (3/2) and Cu2p (Cu (II)), respectively, which are typical for CuO NPs [45]. Additionally, the presence of Cu+ (Cu(I)) was observed, illustrating the partial reduction of Cu^2+^. Both spectra demonstrated the presence of mixed oxidation states, a phenomenon typical of copper oxide nanomaterials owing to their susceptibility to air and moisture during handling and processing. Moreover, the shakeup satellites are characteristic of materials having a d9 configuration in the ground state, i.e., Cu^2+^. Under X-ray irradiation, Cu^2+^ is known to undergo reduction. The reduction is usually associated with the formation of Cu^+^; however, it is also possible that complete reduction to Cu^0^ occurs, but the metal at its basal state (i.e., Cu^0^) cannot be distinguished from Cu^+^ by XPS because of their spectral overlap [44,45,46].

Furthermore, FTIR spectra were recorded at room temperature to evaluate the structural and chemical nature of CuO and Cu_2_O NPs (Figure 1c and Figure 1d, respectively). For CuO NPs (Figure 1c), the two most prominent and characteristic bands were observed around 502 and 596 cm^−1^. These bands could be attributed to the A_u_ and B_u_ modes and assigned to the CuO stretching vibrations (along [101] direction).

**Figure 2:**
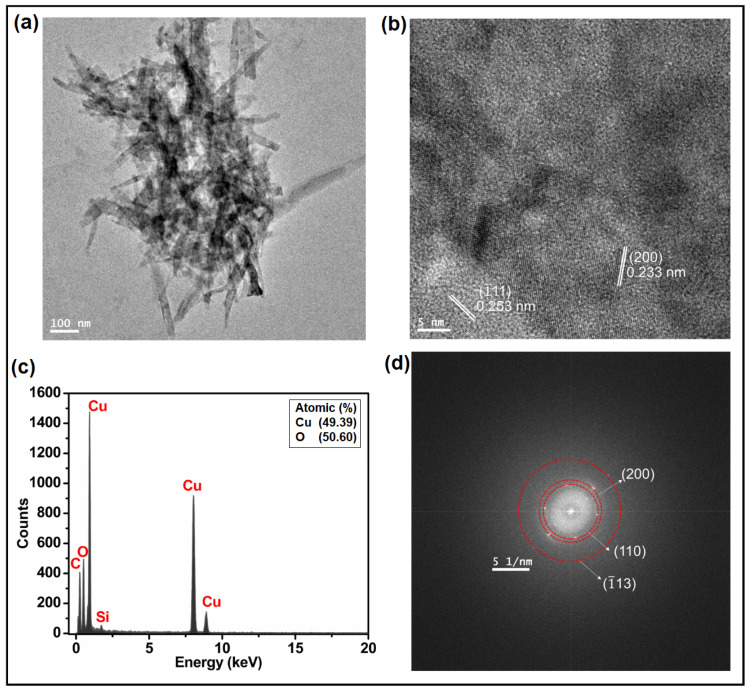
(**a**) TEM image, (**b**) HRTEM image, (**c**) EDX spectrum, and (**d**) SAED of CuO NPs.

Additionally, no IR signals were identified in the 605 to 660 cm^−1^ range, thereby ruling out the possibility of another phase, such as Cu_2_O NPs. A significant broad band was observed in the 3200 to 3550 cm^−1^ range, which could be attributed to the stretching vibration of hydroxyl groups (-OH) due to the adsorption of water molecules on the surface of the NPs [43]. The bands corresponding to the in-plane bending (793 cm^−1^), out-of-plane bending (1081 cm^−1^), symmetric stretching (1137 cm^−1^), and asymmetric stretching (1421 cm^−1^) of the C-O bonds in the carbonate ion (CO_3_^2−^) were also observed, leading to the hypothesis that a thin layer of carbonate is formed over the surface of the CuO NPs in air [47]. The IR bands detected in the regions mentioned also confirmed the construction of single-phase CuO NPs with monoclinic structures. In the FTIR spectrum of Cu_2_O NPs, the strong peak at 603 cm^−1^ illustrated the Cu-O stretching band [32]. The peaks observed at 1081, 1405, 2924, and 3304 cm^−1^ could be attributed to the C-C stretching, O-H bending, C-H stretching, and O-H stretching vibrations of glycerol, respectively. Due to the inherent viscosity of glycerol, it remained in the developed nanomaterials even after multiple washings and extended drying periods. UV-vis spectroscopy also confirmed the synthesis of CuO NPs and Cu_2_O NPs (Figure 2c and Figure 2d, respectively). In the UV-vis spectrum of CuO NPs, a strong absorption peak can be observed at 325 nm corresponding to the surface plasmon resonance (SPR) (Figure 1e). This absorption peak could be attributed to the oscillation of surface conduction electrons, which become stimulated by the incoming electromagnetic radiation [48].

**Figure 3:**
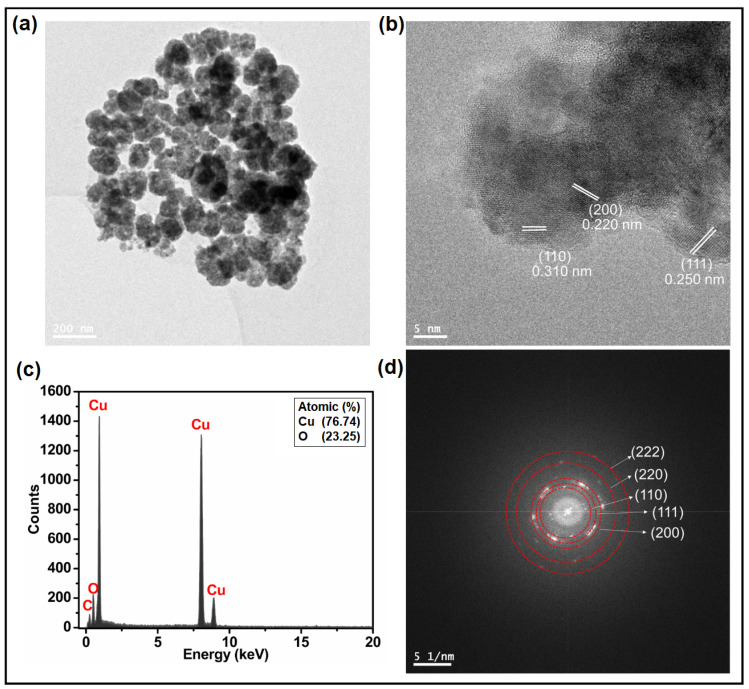
(**a**) TEM image, (**b**) HRTEM image, (**c**) EDX spectrum, and (**d**) SAED of Cu_2_O NPs.

The UV-vis spectrum of Cu_2_O NPs showed a broad adsorption peak centred around 500 nm (Figure 1f) [49]. The low-magnification TEM image of CuO and Cu_2_O NPs (Figure 2a and Figure 3a) clearly demonstrates the formation of needle-shaped CuO NPs and spherical Cu_2_O NPs, respectively. Using different precursors, i.e., NaOH (for CuO NPs obtention) and glycerol (for Cu_2_O NPs obtention), during the sonochemical synthesis resulted in different NPs morphologies. The HR-TEM images of CuO NPs (Figure 2b) corroborate the exposure of $(\bar{1}$10) and (200) facets with a d-spacing of 0.253 and 0.233 nm, respectively. These values were in good correlation with the d-spacing obtained from XRD results (Table S1, Supplementary Material). The HR-TEM results of Cu_2_O NPs (Figure 3b) support the exposure of (110), (111), and (200) facets with a d-spacing of 0.310, 0.250, and 0.220 nm, respectively. These values were similar to those obtained from the XRD results (Table S1, Supplementary Material). Finally, the EDX spectra (Figure 2c and Figure 3c) confirmed the presence of copper (Cu) and oxygen (O) in both CuO and Cu_2_O NPs. However, the ratio of the difference in the heights of Cu and O peaks is almost double in the case of Cu_2_O NPs compared to CuO NPs, confirming the stoichiometric ratios of Cu and O in these NPs. The selected area electron diffraction (SAED) pattern rings of CuO NPs (Figure 2d) displayed three rings indexed to the (110), (200), and ($\bar{1}$13) planes. Similarly, the SAED pattern rings of Cu_2_O NPs (Figure 3d) correspond to the exposed (110), (200), (111), (220), and (222) planes.

### 3.2. Antimicrobial Activity of CuO and Cu_2_O NPs

**Figure 4:**
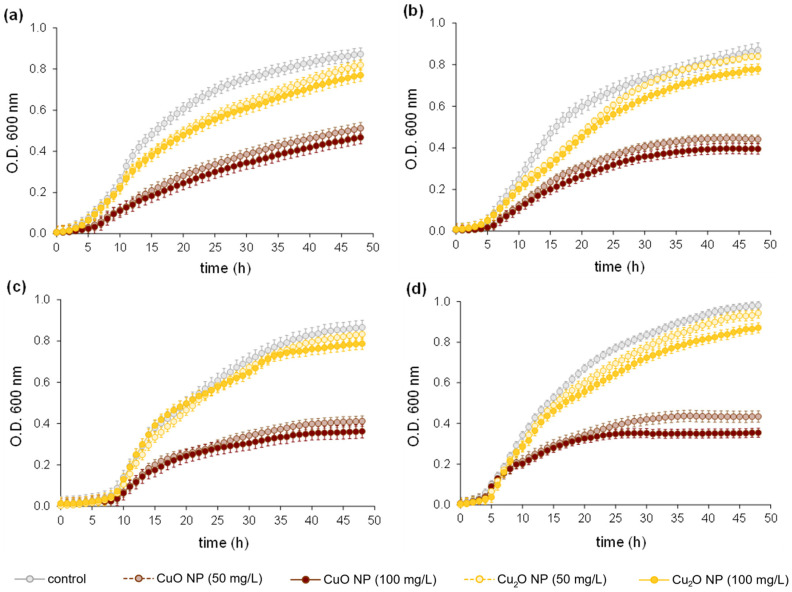
Effect of CuO and Cu_2_O NPs on the growth of the copper-resistant (**a**) *P. lactis* UKR1, (**b**) *P. panacis* UKR2, (**c**) *P. veronii* UKR3, and (**d**) *P. veronii* UKR4 strains.

The copper-resistant UKR strains showed similar behaviours against both CuO and Cu_2_O NPs (Figure 4). In all cases, CuO NPs were more efficient than Cu_2_O NPs in inhibiting bacterial reproduction in LB medium. A clear bacteriostatic effect was observed for the CuO NPs at the concentrations tested (i.e., 50 and 100 mg/L); meanwhile, a slight decrease in the OD_600_ values during the first 15–20 h was observed for UKR1 and UKR2 growing in the presence of 50 and 100 mg/L of Cu_2_O NPs. On the contrary, such differences were not observed in the growth kinetics of both P. veronii strains (UKR3 and UKR4), considering the control condition and the presence of Cu_2_O NPs (Figure 4c,d). Nevertheless, accounting for the lower antimicrobial activity revealed by Cu_2_O NPs compared with CuO NPs, the OD_600_ values reached by all bacteria growing at 100 mg/L of Cu_2_O NPs were between 10 and 15% lower than those in the controls.

On the other hand, no substantial differences in the growth profile of the UKR strains were observed at doubling NPs concentration from 50 to 100 mg/L. This behaviour suggests the action of Cu_2_O and CuO NPs against the tested *Pseudomonas* is not linearly dependent on the concentration. Noticeably, the OD_600_ values reached by the UKR strains growing in the presence of 50 and 100 mg/L of CuO NPs were lower than the ones reached with 500 mg/L Cu(II) (Figure S1, Supplementary Material), proving the greater bacteriostatic effect achieved by these NPs and the high bacterial resistance towards the free metal ion (i.e., Cu^2+^).

**Figure 5:**
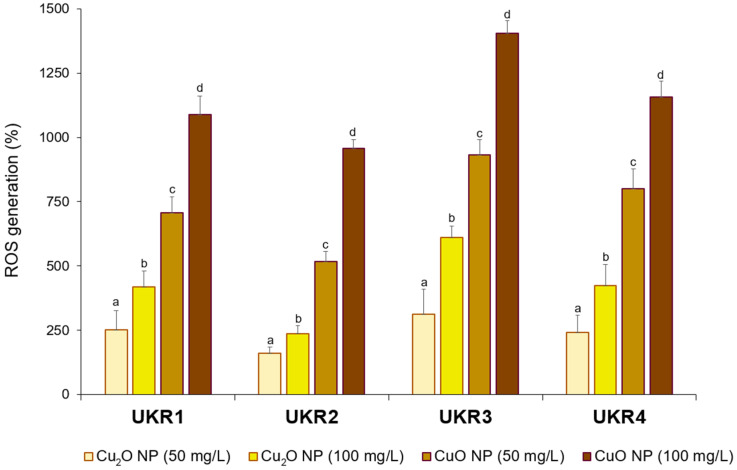
Reactive oxygen species (ROS) generation in copper-resistant (a) *P. lactis* UKR1, (b) *P. panacis* UKR2, (c) *P. veronii* UKR3, and (d) *P. veronii* UKR4 treated with Cu_2_O or CuO NPs. Different letters represent statistically significant differences (*p* < 0.05) between NPs type and concentration for each strain; e.g., “a” is different from “b”.

It has been reported that one of the main mechanisms involved in copper oxide NP action includes the generation of oxidative stress [29] as well as the tendency of these NPs to alternate between the Cu(I) and Cu(II) oxidation states of the metal [18,50]. Comparatively, CuO NPs induced higher oxidative stress (i.e., a larger amount of ROS) in the UKR strains during the first 60 min of contact than Cu_2_O NPs (Figure 5). The response of each strain was also different in terms of the amount of ROS generated, probably because of the expected metabolic divergences between them.

More interestingly, the results from Figure 5 are especially attractive when considering that ROS are not only responsible for deleterious effects in bacterial cells but also participate in cellular signalling. ROS are involved in metabolic processes related to cells’ tolerance against different stressors, including the presence of toxic metals [51,52]. Thus, the lower amount of ROS formed during UKR strain treatment with Cu_2_O NPs could partially explain their more conserved (i.e., less affected) growth kinetics (Figure 4).

On the other hand, several reports have confirmed that all copper oxide NPs tend to release free metal ions [20,21,22], and part of their bactericidal effect is related to the oxidation states of the metal [18,50]. To evaluate Cu_2_O NP stability, the well-established colorimetric method based on the complexation reaction between 4-(2-pyridylazo)resorcinol (PAR) and cupric ions (Cu^2+^) (detection limit 0.1 mg/L) was used to determine the presence of the free metal in the medium containing Cu_2_O NPs [53]. Notably, Cu^2+^ (~15 and ~35 mg Cu^2+^/L for 50 and 100 mg/L Cu_2_O NPs added to the sample) was detected in LB supplemented with Cu_2_O NPs, confirming the tendency of the cuprous cation (Cu^+^) to be released from the NPs and oxidised to the cupric state (Cu^2+^) in such medium. Copper (I) has been reported to be considerably more toxic than copper (II) [54]. Therefore, the above results could explain the lower cytotoxicity observed for the Cu_2_O NPs used in this work because of their partial conversion to Cu^2+^. Considering that the *Pseudomonas* tested here proved to be resistant to a high-free Cu^2+^ concentration [29] (Figure S1, Supplementary Material), the partial instability of the Cu_2_O NPs (i.e., release of cupric ions) seems to be one of the factors explaining their lower capacity to inhibit bacterial growth and induce oxidative stress in the copper-resistant UKR strains (Figure 4 and Figure 5).

Related to this, in a recent study, Bezza et al. [18] synthesised Cu_2_O NPs in reverse micellar templates by using a lipopeptide biosurfactant as a stabilising agent. The developed NPs demonstrated robust antibacterial activity against *Bacillus subtills* and *P. aeruginosa* at 65 mg/L and pH = 5, suggesting that the presence of the lipopeptide biosurfactant improved Cu_2_O NPs’ bactericidal effect. However, at neutral pH (i.e., pH = 7), no significant antimicrobial activity (measured as OD_600_ values) was observed for these NPs up to 125 mg/L. Our results agree with these previous observations, suggesting that higher Cu_2_O NP concentrations than the one tested here could be needed to achieve higher bactericidal performance under physiological pH values (such as the one used in this study), even in the case of stabilised Cu_2_O NPs. However, the use of a high concentration of metal NPs is frequently associated with environmental concerns. Therefore, an equilibrium between maximal antimicrobial activity and minimal NP doses is desirable.

On the other hand, CuO NPs are characterised for being more chemically stable and having a longer shelf life in different media compared to Cu_2_O NPs [20]_._ Using the same colorimetric approach based on the high reactivity of PAR with cupric ions, we attempted to quantitate free Cu^2+^ potentially released from CuO NPs to the medium, but the detection was fully masked by the strong complexation capacity of PAR (high-equilibrium constant) that completely displaced the reaction to the complex formation. However, given the higher bacteriostatic activity shown by these NPs compared to the Cu^2+^-resistant profile of the UKR strains (Figure S1, Supplementary Material), it seems clear that the antimicrobial activity of CuO NPs is dominated by the conservation of their structure.

### 3.3. TEM and SEM Ultrastructure Analysis

**Figure 6:**
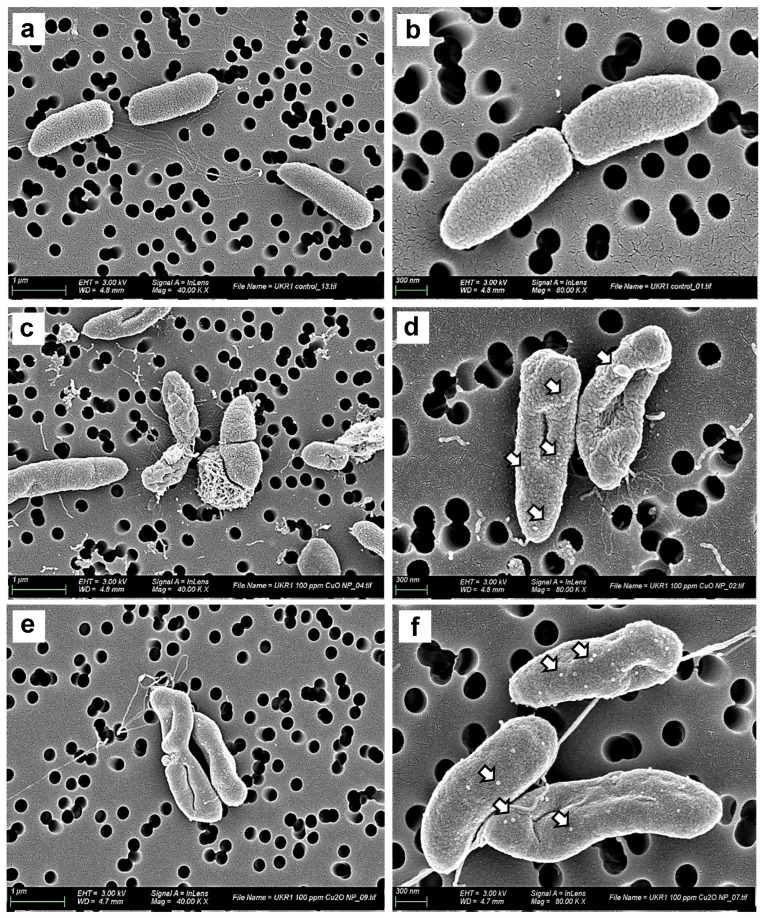
Representative SEM micrographs of untreated *P. lactis* UKR1 cells (**a**,**b**), 100 mg/L CuO NPs-treated cells (**c**,**d**), and 100 mg/L Cu_2_O NPs-treated cells (**e**,**f**). NP-treated cells show straightforward evidence of membrane injury, cytoplasmic leakage, and cell morphology alteration. White arrows indicate vesicle formation on the bacterial surface.

To further elucidate the antimicrobial mechanisms underlying the action of CuO and Cu_2_O NPs on the UKR strains, we examined the bacterial cell morphology and cellular ultrastructure after NPs treatment. SEM observation was carried out on the *P. lactis* UKR1 strain (used as the test model bacteria) to visualise the morphological changes on the bacterial membranes treated with 100 mg/L of Cu_2_O or CuO NPs (Figure 6). Clear differences can be seen in the morphology of the control untreated cells and the NP-treated bacteria. The control cell membranes remained conserved and uniformly rounded (Figure 6a,b), typical of Gram-negative rod-shape bacteria such as *Pseudomonas*. While *P. lactis* UKR1 treated with both copper oxide NPs showed membrane disruption and withered morphology with leakage of intracellular content, consistent with cell damage and lysis (Figure 6c–f). The detrimental effects were also more pronounced after treatment with CuO NPs (Figure 6c,d), which agrees with their higher antimicrobial effect (Figure 4).

A distinct feature observed in *P. lactis* UKR1 cells treated with copper oxide NPs was the formation of outer membrane vesicles that protruded from the bacterial cell surface (Figure 6d–f, white arrows). Membrane vesicles are nano-sized membrane-originated vesicles produced by pathogenic bacteria as a defensive mechanism to combat different stressors. These structures alleviate the destructive effects of antibiotics or other types of antibacterial treatments [55]. Remarkably, membrane vesicle production seems to be higher in UKR1 cells treated with Cu_2_O NPs (Figure 6d). Hence, given that hypervesiculation may affect the activities of antibacterial agents as well as increase bacteria adaptabilities to stress-inducing factors, these observations are in line with the lower activity shown by the Cu_2_O NPs against UKR strains (Figure 4 and Figure 5). Additionally, membrane vesiculation is also induced under oxidative stress [56]. Outer membrane modifications constitute a defensive mechanism against oxidative damage and can result in bacteria hypervesiculation [57]. For instance, an increased production of membrane vesicles in *P. aeruginosa* PAO1 as a response to free radicals has been shown [58]. Our results (Figure 6) agree with this previous report, since the observed ROS formation induced by copper oxide NPs could trigger the production of membrane vesiculation in the wild-type *Pseudomonas* UKR strains.

### 3.4. Anti-Biofilm and Anti-Biofouling Activity of Copper Oxide NPs against UKR Strains

Antimicrobial-resistant bacteria represent a worldwide problem. As previously stated, the four novel copper-resistant UKR isolates (*P. lactis* UKR1, *P. panacis* UKR2, *P. veronii* UKR3, and *P. veronii* UKR4) also express antibiotic-resistant genes [29]. Resistance development is facilitated by bacteria’s biofilm-forming ability, which protects them by enclosing the pathogenic microorganism in a complex extracellular matrix. Accordingly, bacteria in biofilms are considerably more resistant to antibiotics and other antimicrobial agents than planktonic cells [5,59,60]. Considering this, we evaluated the ability of Cu_2_O and CuO NPs to affect the biofilm-forming capacity of the resistant UKR strains.

**Figure 7:**
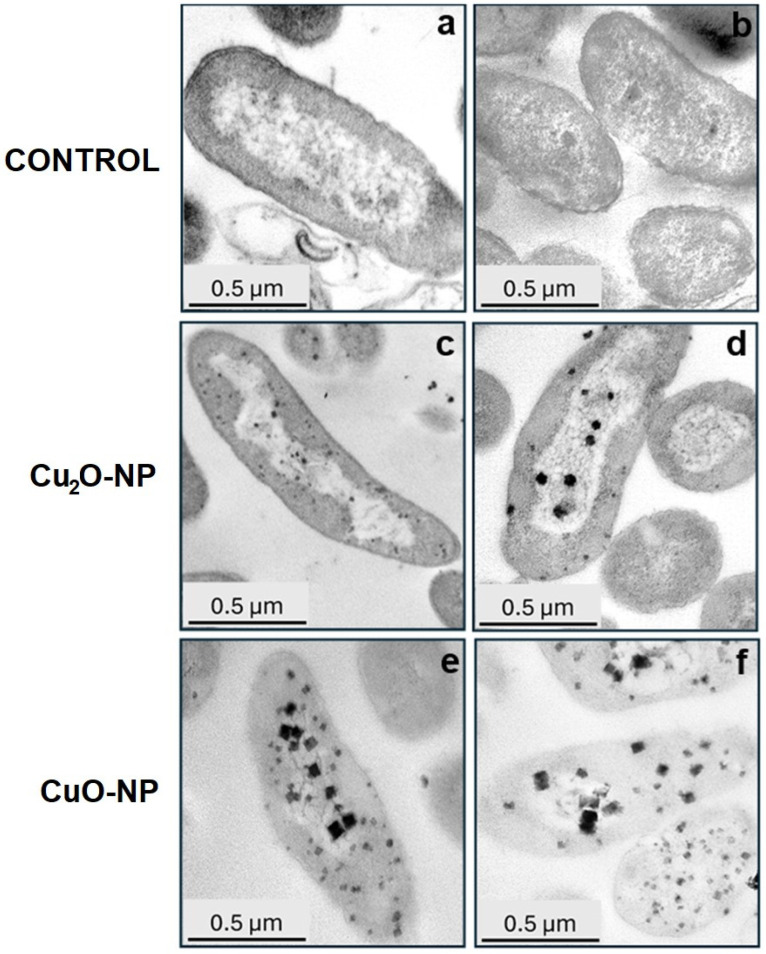
Representative TEM micrographs of untreated *P. lactis* UKR1 cells (**a**,**b**), 100 mg/L Cu_2_O NP (**c**,**d**), and 100 mg/L CuO NP-treated cells (**e**,**f**). A considerable number of intracellular nanoparticles attached to the bacterial cells’ surface (black regular forms) can be observed in Cu_2_O and CuO-treated bacteria. The scale bar represents 0.5 µm.

**Figure 8:**
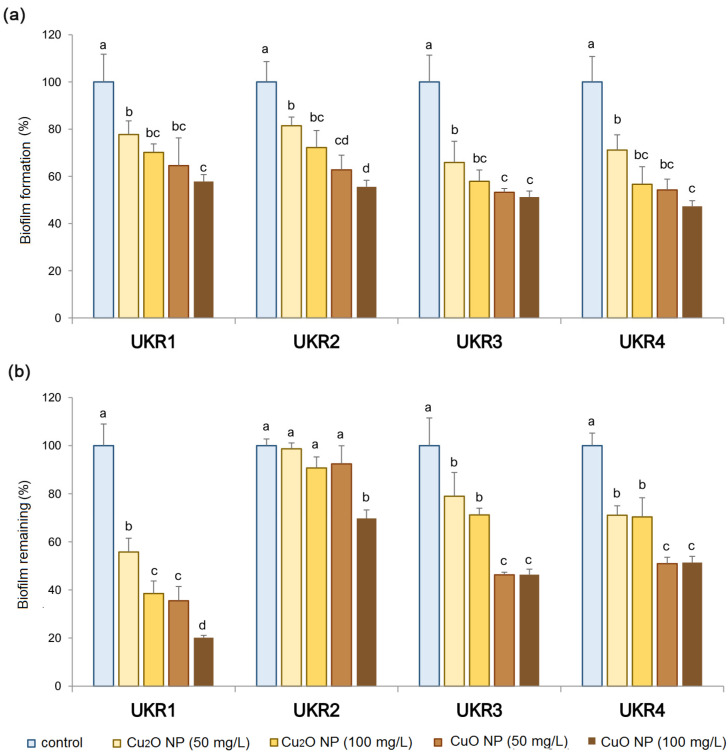
Quantification of biofilm formation (**a**) and bacterial biofilm remaining (**b**) after 48-h treatment in the absence (control) and presence of 50 mg/L and 100 mg/L Cu_2_O or CuO NPs. Error bars indicate standard deviations (S.D.). Different letters represent statistically significant differences (*p* < 0.05) between NPs type and concentration for each strain; e.g., “b” is different from “c” but not from “bc”.

To complement the results of SEM, the ultrastructure of *P. lactis* UKR 1 cells treated with Cu_2_O or CuO NPs was examined by TEM. Micrographs of the untreated bacterium showed rod-shaped cells and presented a uniform electron density, suggesting a normal condition (Figure 7a,b). After exposure to Cu_2_O NPs and CuO NPs (Figure 7b,c,e,f), a large number of NPs were detected inside bacteria and attached to the cell wall. In addition, the TEM images showed prevalent low density in the CuO NP-treated UKR1 cells (Figure 7e,f), suggesting more severe cytoplasmic damage, membrane integrity loss, and cytoplasm leakage, corroborating the SEM results (Figure 6) and confirming the more serious damage on the UKR strain upon exposure to 100 mg/L of CuO NPs (Figure 4, Figure 5 and Figure 6). Accordingly, both TEM and SEM micrographs positively demonstrate that copper oxide NPs came into contact with bacterial cell membranes, which in turn can disrupt the respiratory system and affect cell viability [23]. Both Cu_2_O and CuO NPs significantly (*p* < 0.05) inhibited the biofilm-forming ability of the UKR strains (Figure 8a). The most significant affection was observed for 100 mg/L CuO NP treatment (~50% reduction compared with the control). The higher antimicrobial activity detected for CuO NPs (Figure 4) would have a corresponding effect on bacterial biofilm establishment. In addition, the results from Figure 6 and Figure 7 also support data from Figure 8a, since conserving membrane integrity and cell morphology play an important role in the capacity of microorganisms for developing biofilm [5,59,60]. Moreover, the nanoparticles’ attachment to the bacterial cell surface could also interfere with the first stage of proper biofilm establishment (i.e., anchoring of the bacteria to the colonising surface) [61]. In addition, the surface adhesion of NPs could also improve their antibiofilm effectiveness by preventing further bacterial aggregation [62]. On the other hand, biofilms are also known to be one of the most hard-to-treat bacteria-associated challenges since microorganisms living in communities are more resistant to antimicrobials. Therefore, to degrade pre-established biofilm is a desirable property of any antimicrobial compound. As can be seen in Figure 8b, the biofilm-degrading ability of the copper-oxide NPs showed dissimilar behaviour depending on the *Pseudomonas* species analysed. For instance, *P. veronii* UKR3 and UKR4 presented similar trends, showing higher biofilm-degrading activity for CuO NPs than Cu_2_O NPs, without significant differences regarding the concentration of NPs tested. Pre-established biofilm from *P. panacis* UKR2 was more resistant to the action of both NPs, while in the case of *P. lactis* UKR1, a higher effect when increasing NP concentration was perceived. Nevertheless, increased biofilm degradation was always observed for all UKR strains when exposed to 100 mg/L of CuO NPs.

There are several reasons for the detachment of bacteria from biofilms, including mechanical disturbances, the production of surfactants (e.g., rhamnolipids in *P. aeruginosa*), the induction of ROS (e.g., Figure 5), the release of exopolysaccharides, and the inhibition of quorum sensing signals [63,64]. Metal-containing NPs can bind to the bacterial surface by electrostatic interactions, which interfere with those between the bacterial cells as well as the attaching surface, thus disrupting and preventing biofilm establishment and growth [65]. Moreover, the shape of the NPs also has an important impact on cell toxicity. It has been reported that NPs undergo a shape-dependent interaction with Gram-negative microorganisms. For example, truncated triangular silver NPs displayed the strongest biocidal action against *E. coli* when compared with spherical and rod-shaped NPs [66]. Our results showed that CuO NPs appear square-shaped when interacting with UKR strains (Figure 7e,f), while Cu_2_O NPs appear to be rounded (Figure 7c,d). Therefore, the mechanism of action of the copper oxide NPs could also be shape-dependent, accounting for the superior antimicrobial and antibiofilm performance observed for CuO NPs (Figure 4 and Figure 8). Another explanation of the NPs’ action is the ability to influence bacterial membrane permeability and interact with different biomolecules (e.g., proteins, lipids, DNA), thus leading to genetic mutations and structural alterations. However, the antibacterial and antibiofilm mechanisms of action of metal-based NPs are not yet fully understood [64,67].

To the best of our knowledge, this is the first report exploring the antimicrobial performance of CuO and Cu_2_O NPs against wild-type Cu^2+^-resistant strains of environmental relevance. Our results suggest that both the metallic oxide (cupric or cuprous) and the species of *Pseudomonas* may influence the bacterial phenotypic and genotypic response, but further studies at a molecular level on the novel UKR strains are required to confirm these hypotheses.

## 4. Conclusions

In this work, a sonochemistry approach was used to produce copper-oxide I (Cu_2_O) and copper-oxide (II)-based NPs (CuO NPs), aimed at controlling novel copper-resistant *Pseudomonas* strains isolated from ecologically diverse environments (Ukrainian, Artic, and Antarctic soils). The antibacterial activity of the CuO NPs was greater compared to the growth inhibition achieved in the four tested *Pseudomonas* (*P. lactis* UKR1, *P. panacis* UKR2, *P. veronii* UKR3, and *P. veronii* UKR4) when treated with similar concentrations of Cu_2_O NP (i.e., 50 and 100 mg/L). However, both copper oxide NPs effectively decreased the biofilm-forming capability of all tested bacteria, most probably due to their ability to interact with the bacterial membrane and affect cell morphology, as revealed by SEM and TEM analyses. On the other hand, Cu^2+^ ions were released, lower ROS levels were triggered, and membrane vesiculation was induced, which seem to be some of the factors involved in the lower antibacterial performance of Cu_2_O NPs against the Cu^2+^-resistant UKR strains. Based on these results, the antibacterial activity of CuO NPs may potentially preclude the spread of metal-tolerant bacteria in the environment. Further studies, including gene expression profiles (e.g., RT-*q*PCR, RNA-Seq), will deepen the understanding of the molecular mechanisms underlying copper oxide-based NPs resistance in copper-tolerant bacteria and contribute to the development of more effective nanobiotechnology-based approaches for eradicating harmful pathogenesis.

## Data Availability

The authors confirm that the data supporting the findings of this study are available within the article.

## Supplementary Materials

The following supporting information can be downloaded at: https://www.mdpi.com/article/10.3390/nano14201644/s1. Figure S1: Growth curves of the copper-resistant (a) *P. lactis* UKR1, (b) *P. panacis* UKR2, (c) *P. veronii* UKR3, and (d) *P. veronii* UKR4 strains in LB medium supplemented with 100 and 500 mg/L of copper sulphate; Figure S2: Schematic representation of Cu_2_O (a) and CuO NPs (b) production by sonochemical synthesis; Figure S3: High-resolution XPS spectra of Cu_2_O (left) and CuO NPs (right); Table S1: XRD parameters of CuO NPs; Table S2: XRD parameters of Cu_2_O NPs.
