# Supplementary material for: Unveiling the Potential of CuO and Cu2O Nanoparticles against Novel Copper-Resistant Pseudomonas Strains: An In-Depth Comparison
Source: Nanomaterials (Basel). 2024 Oct 13;14(20):1644. doi: 10.3390/nano14201644 (PMC11510091; doi:10.3390/nano14201644)
Supplement: Supplementary file 1 [file nanomaterials-14-01644-s001.zip › nanomaterials-3232764-supplementary.pdf]

nanomaterials

MDPI

Supplementary material

Unveiling the Potential of CuO and Cu_2O Nanoparticles Against Novel Copper-Resistant *Pseudomonas* strains: An In-depth Comparison

Olesia Havryliuk [1,2], Garima Rathee [3], Jeniffer Blair [3], Vira Hovorukha [1,4], Oleksandr Tashyrev [1], Jordi Morató [2], Leonardo M. Pérez [2,3]*, and Tzanko Tzanov [3]

1 Department of Extremophilic Microorganisms Biology, D. K. Zabolotny Institute of Microbiology and Virology of the National Academy of Sciences of Ukraine, 154 Zabolotny St., 03143 Kyiv, Ukraine; gav_olesya@ukr.net or olesia.havryliuk@upc.edu (O.H.); vira-govorukha@ukr.net or vira.hovorukha@uni.opole.pl (V.H.); tach2007@ukr.net or oleksandr.tashyrev@uni.opole.pl (O.T.)
2 Laboratory of Sanitary and Environmental Microbiology (MSMLab), UNESCO Chair on Sustainability, Universitat Politècnica de Catalunya-BarcelonaTech (UPC), Rambla de Sant Nebridi 22, 08222 Terrassa, Barcelona, Spain; jordi.morato@upc.edu (J.M.); leonardo.martin.perez@upc.edu (L.M.P.)
3 Grup de Biotecnologia Molecular i Industrial, Departament d'Enginyeria Química, Universitat Politècnica de Catalunya-BarcelonaTech (UPC), Rambla de Sant Nebridi 22, 08222 Terrassa, Barcelona, Spain; garima.rathee@upc.edu (G.R.); jeniffer.blair@upc.edu (J.B.)
4 Institute of Environmental Engineering and Biotechnology, University of Opole, 45-040 Opole, Poland
* Correspondence: tzanko.tzanov@upc.ed

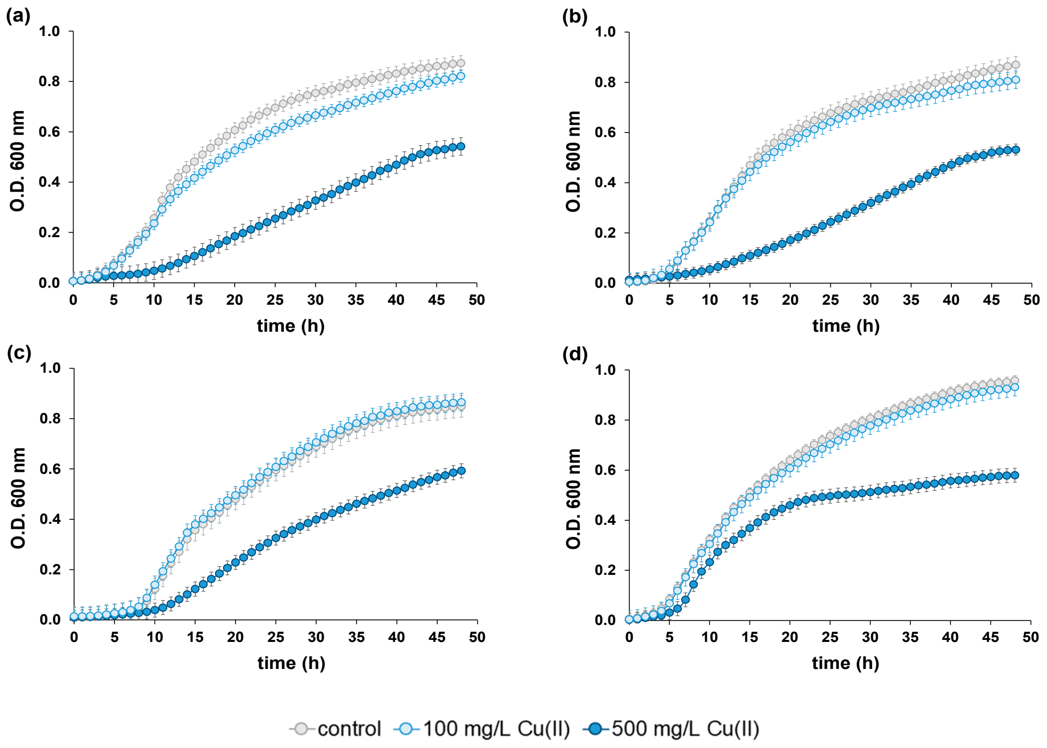

Figure S1. Growth curves of the copper-resistant (a) *P. lactis* UKR1, (b) *P. panacis* UKR2, (c) *P. veronii* UKR3, and (d) *P. veronii* UKR4 strains in LB medium supplemented with 100 and 500 mg/L of copper sulphate.

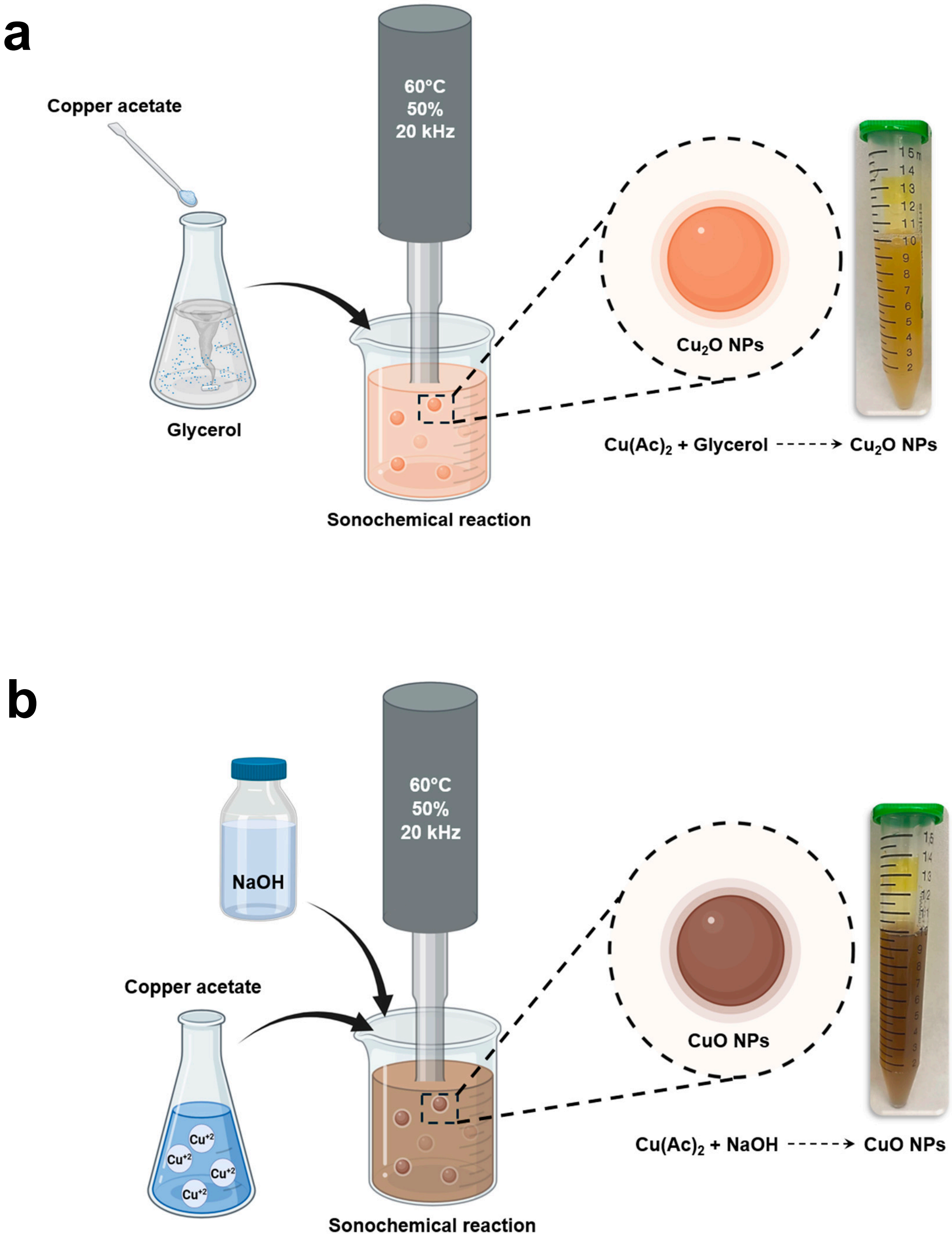

Figure S2. Schematic representation of Cu_2O (a) and CuO NPs (b) production by sonochemical synthesis.

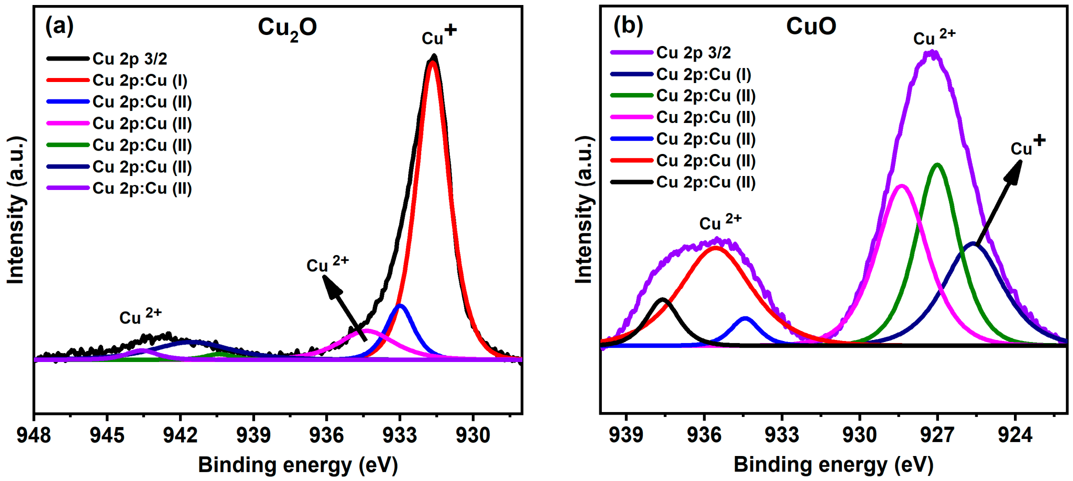

Figure S3. High-resolution XPS spectra of Cu_2O (left) and CuO NPs (right).

Table S1. XRD parameters of CuO NPs

h	k	l	2θ/deg	d-spacing/nm
1	1	0	32.14	0.278
$\bar{1}$	1	1	35.19	0.255
2	0	0	38.35	0.234
$\bar{2}$	0	2	48.45	0.188
0	2	0	53.06	0.172
2	0	2	57.70	0.159
$\bar{1}$	1	3	61.12	0.152
0	2	2	65.85	0.145
2	2	0	67.64	0.138
3	1	1	71.87	0.131
2	2	2	74.59	0.127

Table S2. XRD parameters of Cu_2O NPs

h	k	l	2θ/deg	d-spacing/nm
1	1	0	29.25	0.305
1	1	1	36.09	0.249
2	0	0	42.03	0.215
2	2	0	61.05	0.152
3	1	1	73.29	0.129
2	2	2	77.16	0.124
